# Persimmon Powder from Discarded Fruits as a Potential Prebiotic to Modulate Gut Microbiota in Postmenopausal Women

**DOI:** 10.3390/foods15030480

**Published:** 2026-01-30

**Authors:** Ester Betoret, Nuria Jiménez-Hernández, Stevens Duarte, Alejandro Artacho, Andrea Bueno, Irene Cruz, Noelia Betoret, María José Gosalbes

**Affiliations:** 1 Instituto Universitario de Ingeniería de Alimentos-FoodUPV, Universitat Politècnica de València, Camino de Vera s/n, 46022 Valencia, Spain; mesbeval@upvnet.upv.es (E.B.); stevensduarte1993@gmail.com (S.D.); acbuebob@etseamn.upv.es (A.B.); 2 Área de Genómica y Salud, Fundación Para el Fomento de la Investigación Sanitaria y Biomédica de la Comunidad Valenciana-Salud Pública, Avenida Cataluña 21, 46020 Valencia, Spain; nuria.jimenez@fisabio.es (N.J.-H.); alejandro.artacho@fisabio.es (A.A.); irenecruz@hotmail.es (I.C.); 3 CIBER de Epidemiología y Salud Pública, Instituto de Salud Carlos III, Monforte de Lemos 3-5, 28029 Madrid, Spain; 4 Facultad de Ciencias de la Salud y Desarrollo Humano, Universidad Ecotec, Km. 13.5 Samborondón, Samborondón 092302, Ecuador

**Keywords:** fruit loss, dehydration, valorization, intestinal simulator, metataxonomics, metagenomics

## Abstract

Faced with the challenge of reducing food waste, transforming discarded fruit into functional ingredients useful for the food industry is a valuable solution. Ingredients from fruit such as persimmons, which are rich in indigestible carbohydrates and bioactive compounds with antiradical capacity, could positively impact on the health of certain population groups due to their potential prebiotic effect. This study aimed to select the most suitable drying conditions and milling intensity for obtaining powdered persimmon ingredients with a prebiotic-like effects observed *in vitro* for postmenopausal women, and to evaluate this effect by considering the stimulation of health-promoting bacterial growth and short-chain fatty acids (SCFAs) production. First, the effect of the drying method (hot air drying at 60 and 70 °C, and freeze-drying) and grinding intensity on antiradical capacity, particle size, and the release of bioactive antiradical components into the intestinal lumen after an *in vitro* gastrointestinal digestion was determined. Next, the effect of these conditions on the microbiota composition of postmenopausal women was preliminary assessed in a batch colonic fermentation experiment for 24 h. The results showed that the ingredient dried with air at 70 °C had the highest phenol and flavonoid content, suffered the least degradation during *in vitro* gastrointestinal digestion and promoted the differential growth of fiber-degrader genera. Consequently, this was the ingredient selected as the most suitable. Lastly, the impact of this ingredient on the microbiota composition of 4 postmenopausal women has been evaluated in a long-term study using the Simulator of the Human Intestinal Microbial Ecosystem (SHIME^®^) coupled to high throughput sequencing. The growth stimulation of health-associated bacteria, such as *Akkermansia muciniphila*, *Faecalibacterium prausnitzii* or *Phascolarctobacterium faecium*, and the promotion of beneficial metabolic pathways, such as the sugar uptake-specific phosphotransferase system, sugar metabolism and propionate and isobutyrate production, were detected along 14 days of persimmon powder supplementation. A holistic framework for promoting human health while advancing environmental sustainability is represented by the combination of sustainable by-product valorization and microbiota-targeted functional food development.

## 1. Introduction

The valorization of the significant amount of food losses and agro-industrial by-products constitutes one of the most significant sustainability challenges facing the global food system. The fruit and vegetable sectors are particularly affected, with large volumes discarded due to processing inefficiencies, seasonable harvests, and strict market standards [1]. The persimmon-based cultivation process entails a generation of waste of up to 20% of its total production, to which must be added the residues from the market. However, this fruit is an excellent source of nutrients and bioactive compounds (polyphenols, carotenoids, vitamins and minerals) that provide significant anti-inflammatory and antioxidant activity [2]. Among the various processes for valorizing these plant-based losses, the dehydration treatment combined with milling is a sustainable alternative for producing functional powdered ingredients, with applications in the food industry and other related areas [3]. The dehydration process reduces water activity, ensuring product stability and extending shelf life, while avoiding the generation of effluents. However, depending on the method of dehydration used, there will be greater or lesser oxidation of the bioactive components and greater or lesser structural damage. These effects will determine the techno-functional properties of the final product [4]. The milling process, on the other hand, increases the versatility and ease of handling of the final powdered ingredients. The intensity of grinding determines the particle size distribution, influencing the bioaccesibility of bioactive compounds as well as the techno-functional properties of the powdered ingredients obtained [5].

Prebiotics are non-digestible food ingredients capable of selectively stimulating the growth or activity of beneficial intestinal microbiota [6]. Moreno-Chamba et al. [7] observed that indigestible carbohydrates from fruits such as persimmons (*Diospyros kaki*) stimulated the growth of colonic bacteria such as *Ruminococcus champanellensis*, *Faecalibacterium prausnitzii*, and *Bacteroides thetaiotaomicron.* In addition, bioactive compounds such as polyphenols also have a beneficial effect on the gut microbiota, in many cases mediated by metabolites such as short-chain fatty acids (SCFAs) [8]. These compounds primarily defined as major fermentation end-products of gut microbiota derived from dietary fibers, have been associated with improved gastrointestinal health, immune modulation, and potential metabolic benefits [9]. In this context, recent research has focused on the development of microbiota-directed foods derived from fruit and vegetable residues, exploiting their inherent carbohydrate and polyphenolic profiles to promote health-associated bacteria and the production of SCFAs [10,11,12].

Menopause is associated with a constellation of physical changes attributable to the loss of estrogen such as hot flashes, bone demineralization and vaginal dryness. Also, an increased incidence of cardiovascular disease, metabolic syndrome and different cancers seem to be associated with both menopause and aging. There is evidence that gut microbiome is involved in steroid biosynthesis and metabolism, thereby affecting testosterone and progesterone systemic levels [13]. Also, altered compositional and functional patterns of the gut microbiome in postmenopausal women have been reported [13,14]. Few studies have addressed the dietary modulation of postmenopausal microbiota. Recently, *in vivo* and *in vitro* studies have shown that the dietary supplementation with isoflavone soy modulates the menopausal woman microbiota, increasing bacteria involved in SCFAs production [15,16].

Based on the above, discarded fruits such as persimmon can be transformed by dehydration and milling to obtain potential prebiotic ingredients that can improve the health of postmenopausal women through gut microbiota modulation.

This study aimed to select the most suitable drying conditions and milling intensity for obtaining powdered persimmon ingredients and to evaluate prebiotic-like effects by considering the stimulation of health-promoting bacterial growth, microbial metabolic activity and SCFA production. Firstly, the persimmon surpluses were transformed into powdered ingredients with high nutritional value applying hot air drying or freeze-drying and milling processes. The effects of operation conditions and particle size on the physicochemical and antioxidant properties of the powder as well as their impact on the postmenopausal woman’s colonic microbiota composition have been determined. The fine and 70 °C air-dried powder was selected and used to evaluate its prebiotic-like effects in a long-term study using the Simulator of the Human Intestinal Microbial Ecosystem (SHIME^®^) coupled to high throughput sequencing techniques. Then, the growth stimulation of health-associated bacteria and the promotion of beneficial metabolic pathways, such as the sugar uptake-specific phosphotransferase system, sugar metabolism and SCFA production, were detected along 14 days of persimmon powder supplementation. Consequently, the combination of sustainable by-product valorization and microbiota-targeted functional food development represents a holistic framework for promoting human health while advancing environmental sustainability.

## 2. Materials and Methods

### 2.1. Raw Material and Processing Conditions

Whole persimmons (*Diospyros kaki* L. var. Rojo Brillante) were supplied directly from the field by the “La Vall de la Casella” organic farming cooperative (Alzira, Spain). After washing the whole fruits under running water, raw material (FP) was crushed at 5000 rpm for 4 s in a TM31 Thermomix food processor (Thermomix^®^ TM6, Vorwerk, Madrid, Spain) and placed in a transverse flow tray dryer (POL-EKO model CLW400 TOP, Controltecnica Instrumentación Científica, S.L, Madrid, Spain) with air at 2 m/s at 60 °C (HA60) or 70 °C (HA70), or lyophilized (LYO) in a freeze dryer (LyoAlfa 6-80, Telstar, Terrasa, Spain). The dried products (HA60, HA70, and LYO) were then ground to obtain a powdered product of two different particle sizes. Half was ground at 10,000 rpm for 2 min at 20 s intervals to produce a fine powder and the other half at 4000 rpm for 20 s at 5 s intervals to produce a coarse powder.

### 2.2. Physicochemical Properties

Water content was determined by drying up 1 g of sample at 60 °C for 24 h at atmospheric pressure followed by in vacuum conditions until reaching stable weight [17].

For water activity measurements, a thin layer of the sample was placed in a dew point hygrometer (Aqualab 4TE; Decagon devices Inc., Pullman, WA, USA) with a sensitivity of ±0.003 at 20 °C.

Brix degrees were measured in a refractometer (AbbeAtago, 3-T, Tokyo, Japan), thermostat at 20 °C. The total soluble solid content was calculated from the moisture content and the Brix degree value.

The total nitrogen content was determined by combustion according to the ISO 16634-1:2008 standard [18] for food products which is based in the Dumas principle. A correction factor of 6.25 was applied to calculate the crude protein content. 

Total, soluble, and insoluble dietary fiber was determined by the official enzymatic-gravimetric method AOAC 985.29.

The particle size of the persimmon powders was determined using the dry method. A Masterizer (Malvern Instruments Limited, Worcester, UK) with a measurement range of 0.02–200 µm and a blue light with a wavelength of 470 nm was used. The particle size distribution was obtained and characterized by the mean equivalent volume diameter (D[3, 4]), the equivalent diameter calculated from the particles’ area (D[2, 3]) and finally d90, d50, and d10, representing the respective percentiles of the distribution, i.e., the volume of particles below 90%, 50% and 10% of those analyzed.

### 2.3. Antioxidant Properties

For extraction of compounds with antiradical capacity, persimmon samples were diluted in the proportion 1:3 (*w*/*v*) with methanol solution 80:20 and kept in stirring for 1 h. They were then centrifuged for 5 min at 10,000 rpm. The antiradical capacity was determined in supernatant. Post-gastric, post-intestinal and post-fermentation samples were centrifuged at 8500 rpm for 15 min as the compounds had already been extracted. The supernatant was then diluted 1:3 (*v*/*v*) with 80:20 (*v*/*v*) methanol solution.

The Folin–Ciocalteu assay [19] was used to analyze total phenolic compounds. For the experiment, 0.125 mL of extract, 0.125 mL of Folin–Ciocalteu reagent and 0.5 mL of distilled water were mixed in the order indicated in a spectrophotometric beaker. After 6 min, 1.25 mL Na_2_CO_3_ 7% (*w*/*v*) in distilled water and 1 mL of distilled water were added. The mixture was allowed to react for 90 min, after which the absorbance was measured at 765 nm in a spectrophotometer (Helios Zeta UV/Vis, Thermo scientific, Wrexham, UK). The results obtained were compared with a reference curve using gallic in the range 0–500 mg/L. The results were expressed as mg equivalent gallic acid (EAG) per g dried sample (mg EAG/g dm).

Flavonoid compounds were determined according to Luximon-Ramma [20] method. The absorbance was measured at 368 nm in a (Helios Zeta UV/Vis, Thermo scientific, Wrexham, UK). Calibration curve was prepared with concentrations of quercetin between 0 and 350 mg/L. Results were expressed as mg equivalents of quercetin per g of dried sample (mg EQ/g dm).

The antiradical capacity was measured according to DPPH scavenging activity assay described by [21]. This method sets to add in a spectrophotometry bucker 0.1 mL of extract, 0.9 mL of pure methanol and 2 mL of methanol-DPPH solution prepared using 100 mM (29.4 mg/mL) of DPPH in methanol. Absorbance was quantified at 0, 30, and 60 min at 517 nm using a spectrophotometer (Thermo Scientific, Helio Zeta U/Vis). The results were expressed as mg trolox equivalents per g dry matter (mg TE/g dm). The trolox calibration curve was used as an antioxidant reference standard, covering a concentration range from 0 to 500 mg/L.

For antiradical capacity determination by ABTS method [22], 0.1 mL of extract was mixed with 2.9 mL of ABTS+ in phosphate-buffered solution (0.7 absorbance at 734 nm) and kept in darkness for 7 min before measuring the absorbance at 734 nm in a spectrophotometer (Thermo Scientific, Helio Zeta U/Vis). A trolox (TE) calibration line, expressed as mg equivalents of trolox per g of dried sample (mg TE/g dm), was used as a reference.

### 2.4. Persimmon Powder In Vitro Simulation of Gastrointestinal Digestion

To simulate gastrointestinal digestion the INFOGEST methodology proposed by Brodkorb et al. [23] was considered. In the static gastrointestinal digestion, a 1:1 ratio (*v*/*v*) of persimmon powders to digestive fluids was maintained. Three replicates were made of each sample, with constant stirring at 37 °C. The products of intestinal digestion were frozen at −80 °C until use.

### 2.5. Fecal Samples and Inoculum Processing

The recruitment of participating women will be carried out at FISABIO. They will be healthy women aged 55–65 with good adherence to the Mediterranean diet and who are not on any hormonal treatment or have received antibiotics, prebiotics, or probiotics in the last 6 months prior to the study.

Participants will be provided with the necessary materials and instructions for collecting fecal samples in a container with a gas generator to create anaerobic conditions. They transported the samples to the laboratory. To prepare the slurries, fecal samples were diluted (30% or 20% *w*/*v*) and homogenized in 0.1 M sterile sodium phosphate buffer at pH 7.0 containing 0.4 g/L cysteine hydrochloride and 50% glycerol as the reducing agent and the cryoprotector, respectively. Finally, after centrifugation to remove the big particles, the fecal slurries were stored at −80 °C until further use in the batch cultures.

The protocol was approved by the Public Health Research Ethics Committee from The Foundation for the Promotion of Health and Biomedical Research of the Valencian Community (FISABIO) (approval number: 20230127/05). All subjects gave their informed consent before they participated in the study.

### 2.6. Batch Colonic Fermentation

The *in vitro* static batch culture fermentation procedure was applied according to [24] with slight modifications. For the fermentation cultures, the basal medium described by [25] was used and the inoculum was added at 5% (*v*/*v*). The predigested powders, HAD60, HAD70, LYO, and FP, were used as the substrate at 5% (*w*/*v*). Moreover, a control fermentation without substrate for each inoculum was performed. The fermentation tubes (10 mL) were incubated at 37 °C in anaerobic jars with oscillating shaking for 24 h. Samples were removed from the fermentation tubes at baseline (t = 0 h) and after 24 h for further analysis.

### 2.7. Dynamic Colon Fermentation with the SHIME^®^ System

To account for inter-individual variability of gut microbiota, the SHIME^®^ system (ProDigest, Ghent, Belgium) was configured with a first reactor simulating the upper gastrointestinal tract, followed by four proximal colon reactors (multi-SHIME^®^ configuration) simulating ascending and transverse colon segments.

After inoculating the colon reactors (250 mL) of the multi-SHIME^®^ system with 5% (*v*/*v*) fecal slurry from four different donors, an overnight incubation was performed. From there, the two-week stabilization period began to allow microbiota to adapt to the *in vitro* environment, followed by a two-week control period, representing the baseline in the different colons. After control period, there was a two-week experimental period. The experimental period started with the addition of 5% (*w*/*v*) digested persimmon fine powder to the SHIME^®^ feed. Following SHIME^®^ protocol, 140 mL of SHIME^®^ medium without starch and 60 mL of pancreatic and biliary fluids were administered to the gastrointestinal compartment three times a day for the duration of all fermentation experiment (6 weeks). The pH was controlled and maintained in the range 5.6–5.9 and the temperature was constantly maintained at 37 °C. All the system was over pressed at 1 bar in anaerobic condition with periodical injections of N_2_. SHIME samples were taken from the four different colonic reactors (A, B, C, D) at different time-points of control (CO) and experimental (EX) periods. Samples were stored at −80 °C until used for analysis of SCFAs and microbiota composition and function.

### 2.8. Total DNA Extraction and Sequencing

Fermentation aliquots were centrifuged, and bacterial cells were recovered for DNA extraction and sequencing. Total DNA from batch cultures was extracted in the robotic workstation MagNA Pure LC Instrument (Roche) using the MagNA Pure LC DNA isolation kit III (Bacteria, Fungi) (Roche Life Science, Basel, Switzerland). Maxwell RSC48 (Promega Biotech Ibérica, Madrid, Spain) extractor was used to purify the total DNA from SHIME samples. DNA samples were quantified with the Qubit dsDNA HS Assay Kit (Thermo Fisher Scientific, UK) and stored at −20 °C until further processing. The V3-V4 hypervariable region of the 16S rRNA gene was amplified using total DNA as a template following the Illumina protocol for 16S Metagenomic Sequencing Library Preparation (Illumina, San Diego, CA, USA). The libraries were constructed following Illumina instructions and sequenced using NextSeq2000 sequencer with the P1 kit (600c) from Illumina (Illumina, San Diego, CA, USA). To construct the metagenomic libraries, the total DNA with the Illumina Nextera^®^XT DNA Sample Preparation Kit (Illumina, San Diego, CA, USA), according to the manufacturer’s instructions was used. NextSeq2000 platform (P1 kit) was used to perform metagenomic sequencing.

### 2.9. Sequence Analysis

The DADA2 R package (version 1.20.0) [26] was employed for sequence read processing and forward and reverse merging as well as to infer Amplicon Sequence Variants (ASVs) with their sample-wise abundances. The variants reconstructed by combining a left-segment and a right-segment from two high abundant sequences, were identified as chimeric and discarded, to obtain the final ASVs. Taxonomic identification was assigned to ASVs using DADA2 and the SILVA v.138 reference database [27]. The MegaBLAST tool from BLAST (v.2.10.0) was further used for those ASVs identified only at genus level, requiring at least 97% identity for species-level assignation and a minimum difference of 2% between the first- and second-best matches.

The metagenomic paired-end reads are filtered out and trimmed with the Fastp application (version 0.23.4) [28]. Human DNA and rRNA sequences were also removed. The cleaned fastq files are analyzed with the SqueezeMeta pipeline (version 1.6.5; database built in September 2023) [29]. For functional annotation, the gene sequences were compared against the Kyoto Encyclopedia of Genes and Genomes (KEGG) database [30] obtaining the gene family identifiers (KO). Tabular summaries are generated from the SqueezeMeta results using the “sqm2tables.py” SqueezeMeta script. The raw counts of KOs and pathways (ko) for each sample were normalized by gene length and sequencing depth giving Transcripts Per Kilobase Million (TPM) normalization.

### 2.10. Microbiota Characterization

Analysis of Composition of Microbiome with Bias Correction (ANCOM-BC2) [31] was used to identify differentially abundant taxa at ASV level between groups, taking in account the inter-individual variability. In addition, a normalized abundance table was generated using ANCOM-BC2 that was used for the beta diversity analysis based on the Bray-Curtis dissimilarity index. Permutational multivariate analysis of variance (PERMANOVA) test was performed using the Bray–Curtis dissimilarity index with the Adonis function from vegan library with 900 permutations, with “strata” parameter to incorporate the individual variability. Bar and longitudinal plots, Principal Coordinates Analysis (PCoA) and canonical correspondence analysis (CCA) were generated with in-house R scripts.

Self-Organizing Maps (SOM) [32] were constructed for taxonomic and functional data sets, using the function ‘som’ from the ‘som’ library in R package. These maps are artificial neural networks that use a neighborhood function to separate a complex, high dimensional input into a reduced number of discrete groups with unique behaviors through time.

Also, it was performed an enrichment analysis that allowed us to identify pathways that are enriched in a gene list more than would be expected by chance. The statistically significant over-represented pathways were represented as network using the R package.

### 2.11. SCFAs Quantification

Short chain fatty acids production was assessed by analyzing acetic, propionic, butyric, isobutyric, valeric and isovaleric acids from the supernatants of the four colonic fermentations (A, B, C, D) in the SHIME^®^ system at different time-points: control period after 14 days (CO14), and after 1 (EX1), 3 (EX3) 7 (EX7), 10 (EX10), and 14 (EX14) days of the experimental period. To improve the detectability of the analytes, the samples were first derivatized. For this purpose, 300 µL of sample were mixed with 500 µL NaOH (5 mM), 500 µL propanol: pyridine (3:2) and 100 µL propyl chloroformate (PCF). The sample was homogenized by vortexing for 1 min and by ultrasound for 1 min and heated at 80 °C for 1 h with stirring at 400 rpm. Extraction was then performed by adding 500 µL of BF3-hexane. Quantification was performed on 200 µL of the resulting supernatant. A mass spectrometer (MS Agilent 5977A, Agilent Technologies Inc, Santa Clara, CA, USA) with low resolution quadrupole analyzer coupled to a capillary gas chromatograph (GC Agilent 7890B, Agilent Technologies Inc, Santa Clara, CA, USA) with automatic injector was used for quantification. Standard calibration curves of acetic (RT 2.3 min), propionic (RT 2.8), isobutyric (RT 4 min), butyric (RT 2.3 min), isovaleric (RT 5.9 min) and valeric acid (RT 6 min) were prepared in the range of 0.1–700,000 µg/L. Data processing and quantification was performed with MS Quantitative Analysis (MS Agilent 5977A, Agilent Technologies Inc., Santa Clara, CA, USA).

### 2.12. Statistical Analysis

The results for analytical determinations were statistically analyzed with Statgraphics software (Centurion XVI.I, Statpoint Technologies, Inc., Warrenton, VA, USA) at a 95% confidence level (*p*-value ≤ 0.05). The normality of the data was tested with the Shapiro–Wilk test (*p*-value > 0.05). The data were processed by simple ANOVA after checking the normality of the data. For each processing treatment, three different experiments with three replicates were carried out. Significant differences (*p*-value ≤ 0.05) among groups were determined by Fisher’s LSD test.

For microbiota analysis, the pairwise comparisons of continuous variables were analyzed using the Wilcoxon rank-sum test. To statistically evaluate differences between groups in continuous variables, the Kruskal-Wallis test was used. The Benjamini–Hochberg procedure was applied for false discovery rate control. To determine the differential ASVs, KOs and SCFAs between control (CO14) and experiment samples (EX1, EX3, EX7, EX10, EX14), it was applied linear mixed-effects (LME) model using the nlme library in R package, establishing the time as second fix effect and the individual as random effect.

## 3. Results

### 3.1. Physicochemical and Antioxidant Properties of Persimmon Powders: Effect of Drying Method and Particle Size

After hot air drying at 70 °C, the moisture content decreased from 79.6% to 5.7%, resulting in a reduction in water activity from 0.86 to 0.26. This value is below the threshold of 0.3 required to ensure total powder stability [33]. The other two dehydration treatments (hot air drying at 60 °C and freeze-drying) were continued for as long as necessary to achieve a similar water activity. Table 1 shows the water, soluble solids, protein, and soluble and insoluble dietary fiber content of dehydrated persimmons. The applied dehydration treatments did not significantly affect the evaluated composition of the powders. Interestingly, the total dietary fiber content was 20.1%, with insoluble fiber accounting for 2.3 times more than soluble fiber.

**Figure 1:**
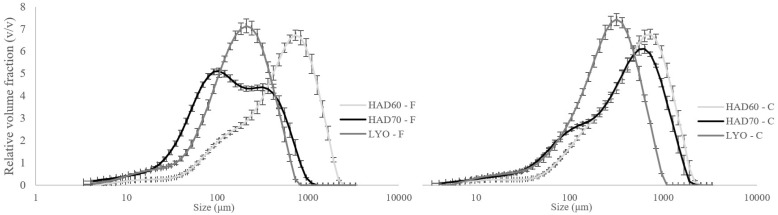
Particle size distribution for fine (F) and coarse (C) persimmon powders obtained from the three dehydration treatments: hot air drying at 60 °C (HAD60), hot air drying at 70 °C (HAD70), and lyophilization (LYO). The lines located at the punctual values in the curves represent the standard deviation from three different measurements.

The drying method had a significant effect on the particle size of the final powders produced under the two grinding conditions. Figure 1 and Table 2 show the particle size distribution and typical distribution parameters for fine (F) and coarse (C) persimmon powders obtained from the three dehydration treatments: hot air drying at 60 °C (HAD60), hot air drying at 70 °C (HAD70), and lyophilization (LYO). The lyophilized samples exhibited a monomodal particle size distribution, whereas the hot-air-dried ones exhibited a less homogeneous distribution with a more pronounced peak at larger particle sizes. However, the effect was different; the more intense grinding conditions aimed at obtaining a fine powder (F) resulted in larger particle sizes for powders dried at 60 °C. These sizes were even larger than those obtained for the less intense grinding conditions (C) at the same drying temperature. Samples dried with hot air, those dried at 70 °C had a smaller particle size than those dried at 60 °C, regardless of grinding intensity.

**Figure 2:**
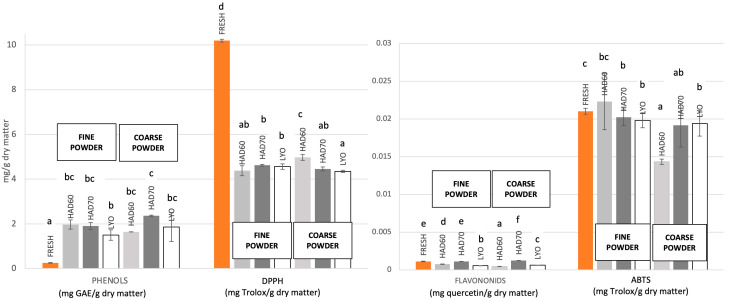
Total phenols and flavonoids content and antiradical capacity determined by DPPH and ABTS methods in fresh persimmon and persimmon powders obtained from the three dehydration treatments: hot air drying at 60 °C (HAD60), hot air drying at 70 °C (HAD70), and lyophilization (LYO). The lines located at the top of each of the bars represent the standard deviation from three different measurements. Different letters indicate significant differences (*p*-value ≤ 0.05).

Moreover, the solubility of the powders, which was found to be between 79% and 85%, considered high for fruit powders, was not significantly affected by the drying method or the grinding conditions. Significant changes were observed in the phenol content, which increased between 6 and 9 times when freeze-drying and grinding to a fine particle size or hot air drying at 70 °C and grinding to a coarse particle size, respectively (Figure 2). This increase can be attributed to the enhanced extractability of phenolic compounds resulting from the breakdown of cellular tissue during freeze-drying and grinding, as well as the conversion of polyphenols in the raw material into more reactive phenolic compounds. Flavonoid compounds did not degrade during hot air drying at 70 °C; in fact, a 10 ± 1% increase was observed in the coarse powder. For the other treatments, degradation of between 32.4 ± 2.3% and 60 ± 4% was observed for hot air drying at 60 °C with fine and coarse particle sizes, respectively. Regarding the effect of processing conditions on antiradical capacity, the results obtained by the DPPH method showed degradation percentages ranging from 51.2 ± 2.6% to 57.4 ± 3.2%, while those obtained by the ABTS method showed percentages ranging from 3.8 ± 0.6% to 31.8 ± 1.2%. For ABTS, the greatest degradation occurred when the sample was dried with hot air at 60 °C and crushed to a coarse particle size. However, crushing the sample to a fine particle size under the same conditions increased the antiradical capacity by 6.1 ± 0.7%.

### 3.2. The Antioxidant Properties of Persimmon Powder Throughout In Vitro Gastrointestinal Digestion

**Figure 3:**
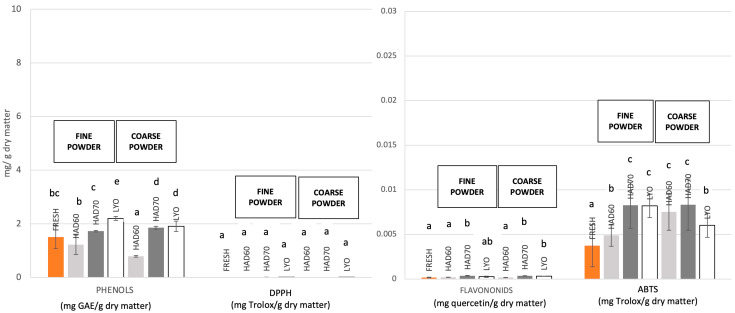
Total phenols and flavonoids content and antiradical capacity determined by DPPH and ABTS methods in fresh persimmon and persimmon powders obtained from the three dehydration treatments: hot air drying at 60 °C (HAD60), hot air drying at 70 °C (HAD70), and lyophilization (LYO) after oral, gastric and intestinal stages of *in vitro* gastrointestinal digestion. The lines located at the top of each of the bars represent the standard deviation from three different measurements. Different letters indicate significant differences (*p*-value ≤ 0.05).

Figure 3 shows total phenols and flavonoids content and antiradical capacity determined by DPPH and ABTS methods in fresh persimmon and persimmon powders after oral, gastric and intestinal stages of *in vitro* gastrointestinal digestion. Practically total degradation of the compounds responsible for the antiradical capacity, as determined by the DPPH method, can be observed in all treatments. The degradation percentage ranges from 53 ± 4% to 80 ± 6% for compounds capable of reacting with the ABTS radical. The lowest degradation percentages were found in coarse powders dried with hot air at 70 °C and in freeze-dried samples with fine particles. However, granulometry did not have a significant effect in these two treatments. The highest degradation percentages were observed in fresh samples and in samples dried with hot air at 60 °C with fine particles. The lowest percentage of degradation in phenols was found ranging from 14.2 ± 1.8% in the powder dried at 70 °C with a fine particle size to 45.2 ± 2.8% in the fine powder dried at 60 °C. The highest degradation in phenol content was found for the powder dried at 60 °C with both particle size, whereas that the hot air drying at 70 °C caused a significantly lower loss of phenols for the powders with fine and coarse particle size. Notably, there is even a positive variation in phenol content in freeze-dried samples.

No differences were found in the degradation of the flavonoids depending on the treatments.

### 3.3. Effects of the Treatments on the Fermentative Microbiota

**Figure 4:**
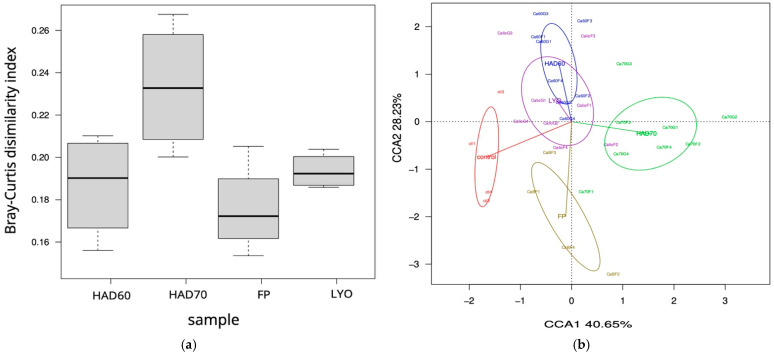
Effect of the drying processes on the fermentative microbiota. (**a**) Bray–Curtis dissimilarity index of the fermentative microbiota growing on the persimmon powders obtained from the three dehydration treatments: hot air drying at 60 °C (HAD60), hot air drying at 70 °C (HAD70), and lyophilization (LYO), respect to control. Kruskal–Wallis test was performed: *p*-value = 0.034, adjusted *p*-value = 0.034. (**b**) Canonical correspondence analysis at ASV level of the bacterial community after fermentations of persimmon powders obtained from the three dehydration treatments: hot air drying at 60 °C (HAD60 or blue), hot air drying at 70 °C (HAD70 or green), and lyophilization (LYO or purple). Control (red) and fresh persimmon (FP or brown).

Since the dehydration and milling processes applied to obtain the powders can affect the composition of the bacterial community growing on them, their effects were assessed by gastrointestinal digestion coupled to a batch colonic fermentation using feces of four women as inoculum. The Bray–Curtis dissimilarity indexes between the microbiota growing on the different substrates (HAD60, HA70, LYO, fresh persimmon (FP)) and the control indicated that HA70 treatment produces a powder that led to a microbiota with the highest dissimilarity (Figure 4). Also, the CCA showed that the powders obtained by the distinct treatments promoted a microbiota with a differential structure (*p*-value = 0.0011) (Figure 4).

**Figure 5:**
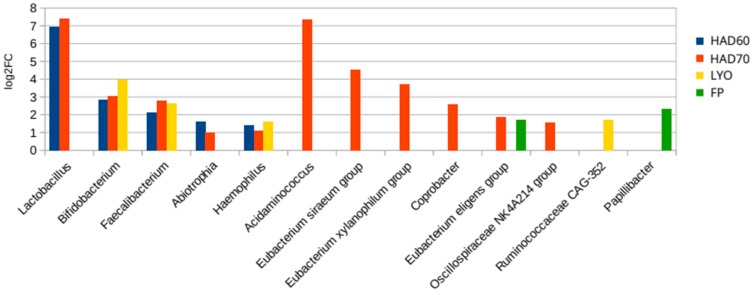
Differential taxa of the fermentative microbiota. Taxa over-represented in the microbiota fermenting the persimmon powders obtained from the three dehydration treatments: hot air drying at 60 °C (HAD60), hot air drying at 70 °C (HAD70) and lyophilization (LYO), and the fresh persimmon (FP). Log2FC, log2 fold change.

To assess the compositional differences between the microbiota growing on the different substrates (HAD60, HAD70, LYO and FP) and the control an ANCOM-BC2 analysis at genus level was performed. Figure 5 represented those taxa that were over-represented in the microbiota fermenting the different powders (*p*-value ≤ 0.05) and presented a log2 fold change (log2FC) higher than 1, highlighting health beneficial bacteria such as *Lactobacillus*, *Bifidobacterium* and *Faecalibacterium.* Moreover, HAD70 treatment promoted the differential growth of fiber-degrader genera belonging to *Eubacteriaceae* family. No significant difference in the microbiota structure was found on the base of the particle size (*p*-value = 0.6).

Overall, drying at 70 °C appears to be the most suitable method for producing a persimmon-based food ingredient. However, a slightly less degradation of the phenol content was observed after gastrointestinal digestion of the powder with a coarse particle size. Instead, batch colonic fermentation revealed that particle size had no effect on the structure of the fermentative microbiota. Thus, due to the operating characteristics of the SHIME dynamic digester, and to prevent blockages in the colon simulator tanks’ feed lines, the powder dried by hot air at 70 °C with a fine particle size was selected to determine its impact on the fermentative microbiota of postmenopausal women in the intestinal simulator (following section).

### 3.4. Compositional and Functional Characterization of the Microbiota from SHIME^®^ Model

**Figure 6:**
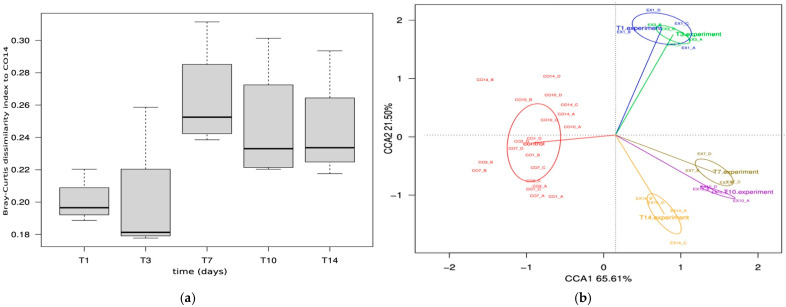
Fermentative microbiota composition during experimental period. (**a**) Bray–Curtis dissimilarity index of the experimental samples respect to T14 of the control (CO14). (**b**) Canonical correspondence analysis at ASV level of the fermentative microbiota during experimental period compared to the control. Red, control samples; blue, day 1 of experimental period (EX1); green, day 3 (EX3); brown, day 7 (EX7); purple, day 10 (EX10); yellow, day14 (EX14).

To assess long-term prebiotic effects of HAD70 powder on the structure and functions of the microbiota, the multi-SHIME^®^ configuration was used. After inoculation of the four colons, two weeks were necessary for microbiota stabilization (Figure S1). To gain insight into microbial composition changes upon persimmon powder addition, firstly the Bray–Curtis dissimilarity index was calculated for the time-points of the experimental period (EX1, EX3, EX7, EX10, EX14) respect to T14 from the control period (CO14) (Figure 6). The highest dissimilarity index was reached after one week of HAD70 persimmon powder administration with a subsequent stabilization. When the time variable was incorporated into a CCA, a progressive change could be observed in the experimental samples. Moreover, the first axis separated the control and experimental samples based on abundance and composition, explaining 65.61% of the compositional variability (Figure 6).

**Figure 7:**
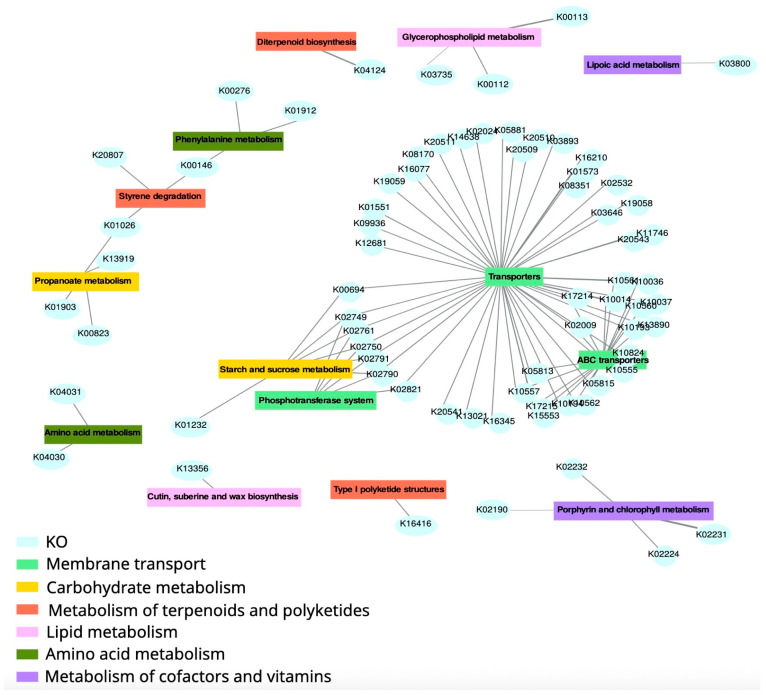
Network of the over-represented pathways in experimental samples. Each metabolic pathways belonged to a functional category.

The dynamics of the fermentative microbiota were determined by means of a SOM analysis. This approach clusters the taxa by similar growth pattern through experimental period (Figure S2). Clusters 1 and 2 included the bacterial taxa that present an increase from CO14 to EX14 time-points. However, while the maximum abundance in cluster 2 occurred after seven days and then remains constant, the maximum in cluster 1 occurred on the third day and subsequently there was a sharp decrease. It was also assessed which bacteria were preferentially growing on persimmon powder applying linear mixed-effects model. Then, those bacteria with an adjusted *p*-value < 0.05 and belonging to SOM cluster 2 were selected, at least in three of the four samples, from the highly abundant phyla, Bacteroidota and Bacillota, and from the minor phyla, Verrucomicrobiota and Deferribacterota (Table S1). Among the 33 selected ASVs, mucin-degrader bacteria such as *Akkermansia muciniphila* and other species belonging to the Muribaculaceae family were detected [34,35]. Five species of *Bacteroides* grew significantly on the persimmon powder. Also, SCFAs-producers such as *Faecalibacterium prausnitzii* presented a differential growth. Moreover, among the bacteria that grow on persimmon powder, it could be pointed out different potential probiotics such as *Phascolarctobacterium faecium*, *Mucispirillum schaedleri* and *Parabacteroides distasonis* [36,37,38]. The growth of three genera of the Lactobacillaceae family (*Lactobacillus*, *Levilactobacillus* and *Ligilactobacillus*) was also detected, as well as the *Christensenellaceae R-7 group*, which has also been described as beneficial to health [39,40,41,42,43]. For the functional composition, the SqueezeMeta pipeline with KEGG database to the metagenome sequences of the different time-points from control (CO1, CO3, CO7, CO10, CO14) and experimental periods (EX1, EX3, EX7, EX10, EX14) was applied. Across all the samples, 7180 KOs and 488 pathways in the metagenome were detected. The whole functional composition was different between the two periods, control and experiment (PERMANOVA, *p*-value = 0.0055) (Figure S3). The CCA showed that the main axis separates the control time-points from those of the experimental period and explains 70.49% of the functional variability detected. By SOM analysis, those KOs that increased their abundance along the experiment period (cluster 0 in Figure S4), were detected. Then, the differential changes in the KOs along the experiment period respect to CO14 time-point using linear mixed-effects model were assessed. The KOs with a *p*-value < 0.01 and belonging to cluster 0 in the SOM analysis, were selected to perform a pathway enrichment analysis. The results showed 14 metabolic pathways that were over-represented in the experiment samples. These pathways belong to the categories: “Membrane transport” (“Transporters” (ko02000), ABC transporter” (ko02010), “Phosphotransferase system (PTS)” (ko02060)); “Carbohydrate metabolism” (Starch and sucrose metabolism (ko00500), Propanoate metabolism (ko00640); “Xenobiotics biodegradation and metabolism” (Styrene degradation (ko00643), Diterpenoid biosynthesis (ko00904), Type I polyketide structures (ko01052); “Amino acid metabolism” (Amino acid metabolism (ko00001), Phenylalanine metabolism (ko00360)); “Lipid metabolism” (Glycerophospholipid metabolism (ko00564), Cutin, suberine and wax biosynthesis (ko00073), “Metabolism of cofactors and vitamins”, (Lipoic acid metabolism (ko00785), Porphyrin and chlorophyll metabolism (ko00860) (Figure 7).

### 3.5. Production of SCFAs

**Figure 8:**
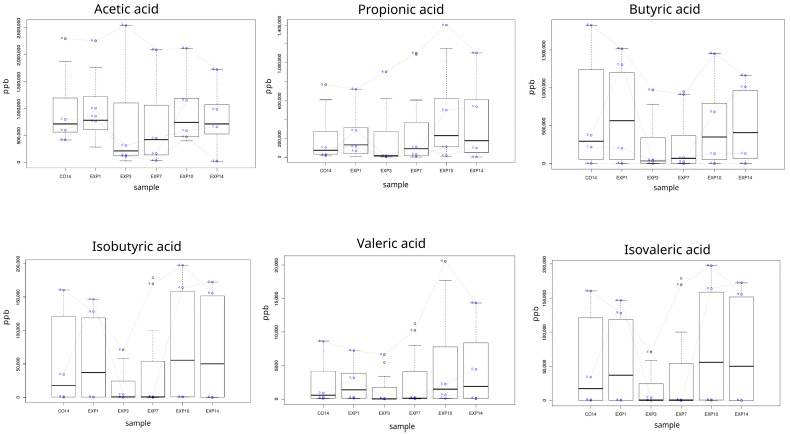
SCFA production (ppb) during 2 weeks of persimmon powder administration. CO14: 14 days of control period, and after 1 (EX1), 3 (EX3) 7 (EX7), 10 (EX10) and 14 (EX14) days of the experimental period. Data are represented as means ± SD (n = 3).

SCFAs are known to be the main bacterial fermentation products from fiber and resistant starch. This study examined the production of SCFAs (acetic, propionic and butyric acids) and branched SCFAs (isobutyric, valeric and isovaleric acids) over a period of two weeks following the administration of persimmon powder. An initial decrease in the production of all SCFAs was observed (Figure 8). The production of SCFA was largely dependent on the individual. Although the fecal sample donors (A, B, C, D)were selected as part of a Mediterranean diet adherence program, SCFA production during the 28 days prior to the powder administration was significantly different, which probably largely conditioned the response to supplementation with persimmon ingredient. However, during the administration period, an increase in the amount of valeric and propionic acids was observed in two of the donors, and an increase in the amount of isobutyric acid in three of them. Moreover, LME model revealed a significant increase in propionic acid *(p*-value = 0.01282, adjust *p*-value = 0.0449) and isobutyric acid (*p*-value = 0.0061, adjust *p*-value = 0.0428) across the experimental period.

## 4. Discussion

The food industry plays a key role in improving the sustainability of food systems, particularly by valorizing agri-food by-products and surplus. Materials derived from fruit and vegetables such as discarded persimmon are especially promising due to their content of bioactive compounds and dietary fiber.

The fiber percentage in dehydrated persimmon was higher than that found in most fresh foods. Plant foods exceeding 20% total dietary fiber include peels, seeds, dried legumes, and bran. Among fruit by-products, dried apple peel has a similar fiber content [44], while orange and mango peels exceed 30% and 40%, respectively [45]. Regarding the ratio between insoluble and soluble fiber, the current authoritative guidance recommends a total adult fiber intake of approximately 25–30 g/day [46] but does not mandate a fixed soluble: insoluble ratio. Clinical practice documents and nutrition education resources commonly advise that a substantial portion of total fiber be soluble, and many practitioners use a practical 2:1 or 3:1 insoluble: soluble rule when formulating diets. In accordance with these recommendations, the persimmon powder obtained has an appropriate proportion of insoluble to soluble dietary fiber. Dietary fiber consists of non-digestible carbohydrates. These non-digestible carbohydrates may be water-soluble, such as pectin, gums and mucilage (soluble dietary fiber), or have a higher molecular weight and be insoluble in water, such as cellulose, lignin and most hemicelluloses (insoluble dietary fiber). The main properties responsible for its beneficial effect on health are its ability to impart viscosity during digestion (in the case of soluble dietary fiber) and its ability to be fermented in the colon by intestinal microbiota (in the case of insoluble dietary fiber) [47,48]. Furthermore, dietary fiber integrated into food matrices imparts technological properties such as gelation, water retention, thickening and structure formation [49]. This would suggest the potential use of persimmon powder as a functional ingredient in food production. Conversely, fiber content can affect the bioaccesibility of active components such as polyphenols. Interactions can occur between indigestible polysaccharide chains and phenolic compounds, resulting in some phenolic compounds becoming insoluble. Thus, they are prevented from being released into the absorbable liquid phase in the small intestine, which limits their bioaccesibility [50].

Another property of powdered foods that affects the digestibility of macromolecules and the bioaccesibility of simple molecules and bioactive compounds is particle size. In general, it can be said that the smaller the particles, the easier it is for mechanical and chemical action to occur during the digestion process, resulting in faster digestion and greater bioaccesibility of nutrients and bioactive compounds [51,52]. Freeze drying involves the sublimation of water under vacuum conditions and at a moderate temperature, which causes a homogeneous breakdown of the cell structure. This results in a monomodal distribution and smaller particle size after grinding. However, hot-air drying causes heterogeneous dehydration and heating of the cell tissue, resulting in phase transitions of different natures that determine the tissue’s heterogeneous resistance to grinding conditions. Consequently, a less homogeneous particle size distribution is produced.

Multiple scientific studies have examined the effect of hot air drying and freeze-drying processes on the antioxidant properties and/or antiradical capacity of plant-based foods such as fruits and vegetables [53]. The results obtained show high variability, largely due to the diversity of the chemical molecules responsible for these properties and their differing stability and extractability when subjected to heat treatment, especially for molecules bound to polysaccharides or structural proteins in the tissue. However, freeze-drying is generally considered to be the dehydration process that best preserves antiradical compounds. In hot air drying, the balance between the degradation of compounds favored by high temperatures, and the generation of others resulting from Maillard reactions, chemical transformations, or structural changes, determines the antiradical capacity of dried products [54,55]. The results of this study demonstrate a substantial impact of the drying process and particle size on the content of phenols, flavonoids, and antiradical capacity. The trends are clearer for phenol and flavonoid content, which is favored when samples are dried with hot air at 70 °C and then crushed to obtain a coarse particle size.

If only the processing results shown in Figure 2 are considered, the samples with the highest phenol content are those dried at 70 °C with coarse particle size, followed by those dried at 70 °C and 60 °C with fine particle size. However, degradation throughout the oral, gastric and intestinal stages (Figure 3) reveals that the samples with the highest phenol content released into the intestinal lumen are freeze-dried samples, followed by coarse-grained samples dried at 70 °C. These results confirm that, while processing conditions affect the nutritional and functional value of food or food ingredients, it is also necessary to consider the digestive process and its effect on the degradation of phenolic compounds during the oral, gastric, and intestinal stages. Furthermore, the impact of these compounds on health depends on how they are transformed in the colon by intestinal microbiota.

Strong evidence supports that dietary fiber, and polyphenols exert highly specific effects on the gut bacterial community, influencing both its composition and function. Furthermore, variations in their chemical structures, such as linkage type, molecular interactions, and degree of polymerization, affect their utilization by the gut microbiota and the subsequent production of secondary metabolites with diverse effects on the host [56,57,58,59]. Since the dehydration treatments affected the physicochemical and nutritional properties of the powdered ingredients, each powder promoted the growth of a microbiota with a differential composition in 24 h colonic fermentations. These results are consistent with other studies [21,24,60]. Moreover, the hot-air drying treatment at 70 °C produced a powder that induced a distinct bacterial community, significantly differing from the control and enhancing the abundance of fiber-degrading and health-promoting bacteria.

Long-term evaluation of HAD70 powder effects revealed an increase in the abundance of *A. muciniphila.* Several studies have shown the modulatory effect of polyphenols on *A. muciniphila* and other keystone species *such* as *B. thetaiotaomicrom*, *F. prausnitzii*, Bifidobacteria, and Lactobacilli [61,62,63,64,65]. *A. muciniphila* utilizes mucin as a substrate, producing acetate and propionate, and promoting cross-feeding interactions with butyrate-producing bacteria [34]. Moreover, low abundance of *A. muciniphila* has been associated with different inflammatory diseases [64,66,67]. *Bacteroides* species also showed a differential growth after persimmon powder supplementation in the SHIME model. *B. thetaiotaomicron*, *B. fragilis*, and *B. massiliensis* have been extensively characterized for their capacity to metabolize polysaccharides and oligosaccharides, thereby supplying nutrients to the host and other commensal microbes [40,65,68]. Specifically, *B. thetaiotaomicron* possesses a wide repertoire of genes encoding carbohydrate-active enzymes (CAZymes), enabling it to degrade complex polysaccharides, including O-linked glycans [69]. Similarly, colonic fermentation of mycoprotein fiber rich in β-glucans has been reported to promote *Bacteroides* growth [70]. In addition to this specialized genus, persimmon powder stimulated the growth of butyrate-producing bacteria such as *Faecalibacterium* and *Ruminococcus*, both associated with host health benefits [71]. Notably, *Faecalibacterium* is considered a biomarker of gut health, as its depletion has been linked to numerous diseases and disorders [72]. Moreover, lactic acid bacteria—well-established probiotics—showed differential growth during the experimental period following persimmon powder supplementation, likely due to their polyphenol-metabolizing capacity [41,42,73]. Additionally, it was also detected an increased abundance of *Phascolarctobacterium faecium*, *Mucispirillum schaedleri* and *Parabacteroides distasonis*, proposed as next-generation probiotics and described as SCFAs-producer, mainly propionate and acetate [36,38,51].

In this work, it has also addressed, by means metagenomics, the functional potential of fermentative microbiota. The majority functional category overrepresented in experimental samples corresponds to membrane transport that included ABC transporters and the sugar uptake-specific phosphotransferase system. Interestingly, ABC transporter represents a system associated with antibiotic resistance. Thus, the increase in these efflux pumps could reveal putative mechanisms for tolerance to the polyphenols in fermentative microbiota. Moreover, the high abundance of “Starch and sucrose metabolism” and “propionate metabolism” pathways after persimmon powder addition suggested a fermentative carbohydrate activity with a differential production of propionic acid. The transient reduction in SCFA production observed in the first 3 days of powder supplementation may be due to bacteriostatic effect of the phenolic compounds in the persimmon powder. A comparable response was reported by García-Villalba et al. [74] following supplementation with a pomegranate extract in a TWIN-SHIME model. Although the production of SCFA was largely dependent on the individual, we observed a significant increase in propionate and isobutyrate after supplementation. Propionic acid has been linked to multiple beneficial health effects, including improved glucose homeostasis, reduced weight gain, and increased satiety [75]. Furthermore, this SCFA exerts neuroprotective and neuroregenerative effects through the gut–brain axis, demonstrating anti-inflammatory and antioxidant activities [76]. *In vitro* cell co-culture studies have also shown that isobutyric acid supports immune function and inhibits tumor growth [77].

Overall, our findings suggest that persimmon surpluses can be explored as a source of high-nutritional-value ingredients through drying and milling processes. The recycling process would not generate effluents or other waste, and the obtained ingredients could be reintroduced into the value chain, thus contributing to the circular economy. However, it would first be necessary to scale up to an industrial level and study possible applications in the food formulation industry. This persimmon-based powder showed prebiotic-like effects in both short- and long-term supplementation, as reflected by changes in the growth of health-associated bacteria and the production of beneficial metabolites in *in vitro* intestinal models. Further nutritional interventions in humans are required to confirm its prebiotic impact on gut microbiota. Moreover, data derived from intestinal simulators must be interpreted with caution due to inherent limitations, particularly the small sample size and the high inter-individual variability of the gut microbiota. Despite this, our results provide proof of concept for reducing food loss and for advancing the development of novel functional foods or microbiota-targeted therapeutics, bringing us one step closer to improving the sustainability of the food system.

## Figures and Tables

**Table 1:** Composition (g/g dry matter) of persimmon after dehydration treatments.

a_w_	0.26 ± 0.03
Water	0.06 ± 0.02 (5.66%)
Soluble solids	0.471 ± 0.015 (44.43%)
Protein	0.034 ± 0.012 (3.21%)
Insoluble dietary fiber	0.149 (14.06%)
Soluble dietary fiber	0.064 (6.04%)

**Table 2:** Typical distribution parameters for fine (F) and coarse (C) persimmon powders obtained from the three dehydration treatments: hot air drying at 60 °C (HAD60), hot air drying at 70 °C (HAD70), and lyophilization (LYO). D[4, 3]: mean diameter of equivalent volume; D[3, 2]: mean diameter of equivalent area; d(0.1), d(0.5), d(0.9): the percentiles of the distribution, i.e., the volume of particles below 90%, 50%, and 10% of the particles analyzed, respectively. Different superscript letters in the same column indicate significant differences (*p*-value ≤ 0.05).

	D [4, 3]	D [3, 2]	d (0.1)	d (0.5)	d (0.9)
HAD60-F	552 ± 23 ^d^	170 ± 6 ^e^	89 ± 2 ^e^	471 ± 22 ^e^	1148 ± 51 ^f^
HAD70-F	193 ± 10 ^b^	64 ± 2 ^a^	33 ± 1 ^a^	128 ± 6 ^a^	456 ± 25 ^b^
LYO-F	189 ± 5 ^b^	80 ± 3 ^b^	43 ± 2 ^b^	161 ± 6 ^b^	378 ± 10 ^a^
HAD60-C	535 ± 19 ^d^	159 ± 3 ^d^	101 ± 1 ^f^	456 ± 19 ^e^	1097 ± 43 ^e^
HAD70-G	71 ± 5 ^a^	114 ± 3 ^c^	58 ± 1 ^c^	353 ± 12 ^d^	937 ± 56 ^d^
LYO-G	270 ± 6 ^c^	112 ± 1 ^c^	61 ± 1 ^d^	235 ± 4 ^c^	531 ± 15 ^c^

## Data Availability

The original contributions presented in this study are included in the article/Supplementary Material. Further inquiries can be directed to the corresponding authors. The sequences generated and analyzed during the current study are available in the EBI database under the project number PRJEB105455.

## Supplementary Materials

The following supporting information can be downloaded at: https://www.mdpi.com/article/10.3390/foods15030480/s1, Figure S1: Bray–Curtis dissimilarity index of the fermentative microbiota respect to the time-point corresponding to 6th day from the start during the stabilization and control periods; Figure S2: Temporal dynamics of bacterial taxa (ASV). The clusters of bacterial taxa are based on their abundance profile throughout experimental period, analyzed by a Self-Organizing Map (SOM) approach. For each cluster, average values at each time-point are shown along with their corresponding 90% confidence intervals, in a scale centered at the mean of all samples and scaled by the standard deviation; Figure S3: Functional composition at KEGG orthologous (KO) level. (a) Principal Coordinates Analysis (PCoA). (b) Canonical Correlation Analysis (CCA); Figure S4: Temporal dynamics of bacterial KOs. The clusters of KOs are based on their abundance profile throughout experimental period, analyzed by a Self-Organizing Map. Table S1: Bacteria with differential growth during the experimental period of SHIME® and belonging to cluster 2 according to the SOM analysis.
