# Supplementary material for: Persimmon Powder from Discarded Fruits as a Potential Prebiotic to Modulate Gut Microbiota in Postmenopausal Women
Source: Foods. 2026 Jan 30;15(3):480. doi: 10.3390/foods15030480 (PMC12897050; doi:10.3390/foods15030480)
Supplement: Supplementary file 1 [file foods-15-00480-s001.zip › Table S1.pdf]

Table S1: Bacteria with differential growth during the experimental period of SHIME® and belonging to cluster 2 according to the SOM analysis

ID	p_value (LMEM)	adjust p_value (LMEM)	Taxonomy	SOM cluster
ASV0624	0.00998119376840943	0.0328547628210144	k__Bacteria;p__Bacteroidota;c__Bacteroidia;o__Bacteroidales;f__Bacteroidaceae;g__Bacteroides;s__acidifaciens	Cluster 2
ASV0162	0.000670921149591876	0.00441689756814652	k__Bacteria;p__Bacteroidota;c__Bacteroidia;o__Bacteroidales;f__Bacteroidaceae;g__Bacteroides;s__coprocola	Cluster 2
ASV0412	0.00011375908591099	0.00105729032787861	k__Bacteria;p__Bacteroidota;c__Bacteroidia;o__Bacteroidales;f__Bacteroidaceae;g__Bacteroides;s__fragilis	Cluster 2
ASV0296	5.60174344829262e-07	1.99633557982794e-05	k__Bacteria;p__Bacteroidota;c__Bacteroidia;o__Bacteroidales;f__Bacteroidaceae;g__Bacteroides;s__massiliensis	Cluster 2
ASV0202	0.0159559838973141	0.0484816433803006	k__Bacteria;p__Bacteroidota;c__Bacteroidia;o__Bacteroidales;f__Bacteroidaceae;g__Bacteroides;s__NA	Cluster 2
ASV0440	0.00235392840831272	0.0106263053860974	k__Bacteria;p__Bacteroidota;c__Bacteroidia;o__Bacteroidales;f__Bacteroidaceae;g__Bacteroides;s__thetaiotaomicron	Cluster 2
ASV0262	0.0056463375949231	0.0217590570731183	k__Bacteria;p__Bacteroidota;c__Bacteroidia;o__Bacteroidales;f__Barnesiellaceae;g__NA;s__NA	Cluster 2
ASV0461	1.74768062637115e-06	3.94476484238059e-05	k__Bacteria;p__Bacteroidota;c__Bacteroidia;o__Bacteroidales;f__Muribaculaceae;g__NA;s__NA	Cluster 2
ASV0362	0.00175903765120254	0.00937506780200756	k__Bacteria;p__Bacteroidota;c__Bacteroidia;o__Bacteroidales;f__Muribaculaceae;g__NA;s__NA	Cluster 2
ASV0389	0.00178007616493814	0.00937506780200756	k__Bacteria;p__Bacteroidota;c__Bacteroidia;o__Bacteroidales;f__Muribaculaceae;g__NA;s__NA	Cluster 2
ASV0499	0.00731022719905397	0.0268608348244309	k__Bacteria;p__Bacteroidota;c__Bacteroidia;o__Bacteroidales;f__Muribaculaceae;g__NA;s__NA	Cluster 2
ASV0222	0.00218095523357061	0.0106263053860974	k__Bacteria;p__Bacteroidota;c__Bacteroidia;o__Bacteroidales;f__Rikenellaceae;g__Alistipes;s__NA	Cluster 2
ASV0665	0.00147280057113756	0.00831080322284765	k__Bacteria;p__Bacteroidota;c__Bacteroidia;o__Bacteroidales;f__Rikenellaceae;g__Rikenellaceae RC9 gut group;s__NA	Cluster 2
ASV0692	1.90372860665388e-05	0.000300789119851313	k__Bacteria;p__Bacteroidota;c__Bacteroidia;o__Bacteroidales;f__Tannerellaceae;g__Parabacteroides;s__distasonis	Cluster 2
ASV0513	1.49405293393257e-08	1.18030181780673e-06	k__Bacteria;p__Deferribacterota;c__Deferribacteres;o__Deferribacterales;f__Deferribacteraceae;g__Mucispirillum;s__schaedleri	Cluster 2
ASV0177	0.00335650254900166	0.0147313167428406	k__Bacteria;p__Bacillota;c__Bacilli;o__Bacillales;f__Planococcaceae;g__Planomicrobium;s__glaciei	Cluster 2
ASV0352	0.00399661093232472	0.0170666088461434	k__Bacteria;p__Bacillota;c__Bacilli;o__Erysipelotrichales;f__Erysipelotrichaceae;g__Faecalibaculum;s__rodentium	Cluster 2
ASV0403	0.00898372987814187	0.0303915197390442	k__Bacteria;p__Bacillota;c__Bacilli;o__Lactobacillales;f__Lactobacillaceae;g__Lactobacillus;s__NA	Cluster 2
ASV0105	0.00110521619386097	0.00665544177841389	k__Bacteria;p__Bacillota;c__Bacilli;o__Lactobacillales;f__Lactobacillaceae;g__Levilactobacillus;s__NA	Cluster 2
ASV0348	4.69196021917063e-09	7.4132971462896e-07	k__Bacteria;p__Bacillota;c__Bacilli;o__Lactobacillales;f__Lactobacillaceae;g__Ligilactobacillus;s__NA	Cluster 2
ASV0520	0.000790821615641642	0.00499799261085518	k__Bacteria;p__Bacillota;c__Clostridia;o__Christensenellales;f__Christensenellaceae;g__Christensenellaceae R-7 group;s__NA	Cluster 2
ASV0546	2.3959919373561e-05	0.000304178273181854	k__Bacteria;p__Bacillota;c__Clostridia;o__Clostridia vadinBB60 group;f__NA;g__NA;s__NA	Cluster 2
ASV0708	6.3409489214163e-07	1.99633557982794e-05	k__Bacteria;p__Bacillota;c__Clostridia;o__Clostridia vadinBB60 group;f__NA;g__NA;s__NA	Cluster 2
ASV0688	0.00225346780961517	0.0106263053860974	k__Bacteria;p__Bacillota;c__Clostridia;o__Clostridia vadinBB60 group;f__NA;g__NA;s__NA	Cluster 2
ASV0882	0.00904051536541188	0.0303915197390442	k__Bacteria;p__Bacillota;c__Clostridia;o__Lachnospirales;f__Lachnospiraceae;g__A2;s__NA	Cluster 2
ASV0239	0.0120122227530894	0.0372143371566299	k__Bacteria;p__Bacillota;c__Clostridia;o__Lachnospirales;f__Lachnospiraceae;g__Blautia;s__obeum	Cluster 2
ASV0377	5.63210447127124e-05	0.000556170316538035	k__Bacteria;p__Bacillota;c__Clostridia;o__Lachnospirales;f__Lachnospiraceae;g__Dorea;s__formicigenerans	Cluster 2
ASV0273	0.00043919028001567	0.00315418473829436	k__Bacteria;p__Bacillota;c__Clostridia;o__Lachnospirales;f__Lachnospiraceae;g__NA;s__NA	Cluster 2
ASV0668	0.000326731063098702	0.00245826228426642	k__Bacteria;p__Bacillota;c__Clostridia;o__Oscillospirales;f__Oscillospiraceae;g__NA;s__NA	Cluster 2
ASV0130	0.00228852938254606	0.0106263053860974	k__Bacteria;p__Bacillota;c__Clostridia;o__Oscillospirales;f__Ruminococcaceae;g__Faecalibacterium;s__prausnitzii	Cluster 2
ASV0005	0.00537457365205906	0.0212295659256333	k__Bacteria;p__Bacillota;c__Negativicutes;o__Acidaminococcales;f__Acidaminococcaceae;g__Phascolarctobacterium;s__faecium	Cluster 2
ASV0064	0.0108467114490142	0.0349751103866171	k__Bacteria;p__Bacillota;c__Negativicutes;o__Veillonellales-Selenomonadales;f__Veillonellaceae;g__Anaeroglobus;s__geminatus	Cluster 2
ASV0078	4.26635334672507e-08	1.53926770035305e-06	k__Bacteria;p__Verrucomicrobiota;c__Verrucomicrobiae;o__Verrucomicrobiales;f__Akkermansiaceae;g__Akkermansia;s__muciniphila	Cluster 2
