# Supplementary figures and images for: Persimmon Powder from Discarded Fruits as a Potential Prebiotic to Modulate Gut Microbiota in Postmenopausal Women
Source: Foods. 2026 Jan 30;15(3):480. doi: 10.3390/foods15030480 (PMC12897050; doi:10.3390/foods15030480)

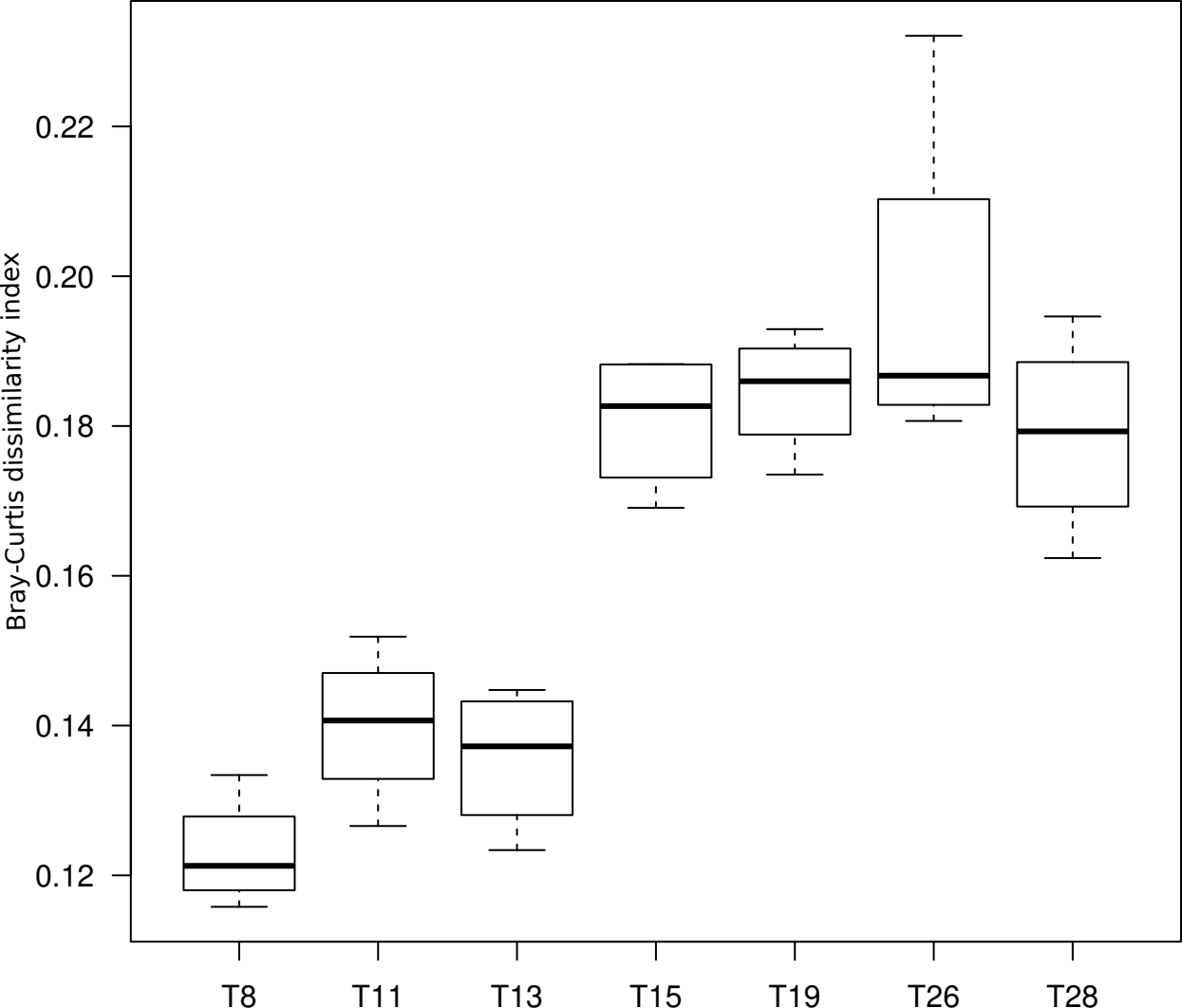

Supplement: Supplementary file 1 [file foods-15-00480-s001.zip › Figure S1.pdf]

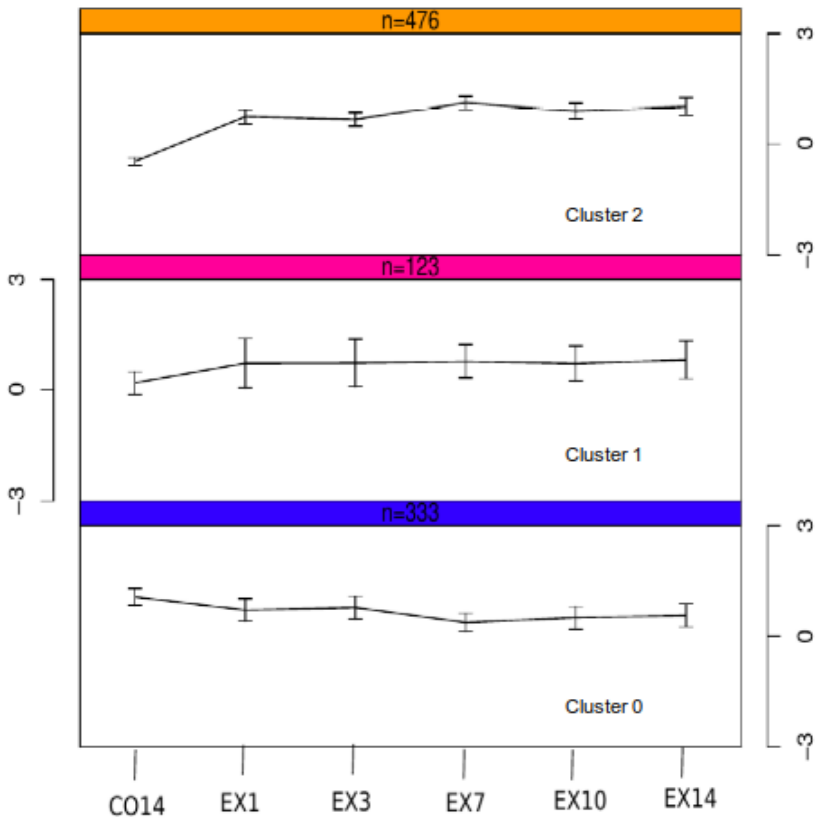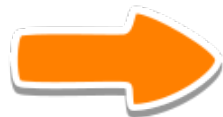

INCREASE

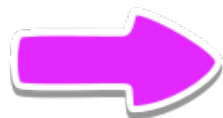

NO CHANGE

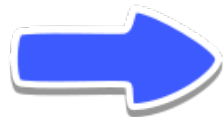

DECREASE

Supplement: Supplementary file 1 [file foods-15-00480-s001.zip › Figure S2.pdf]

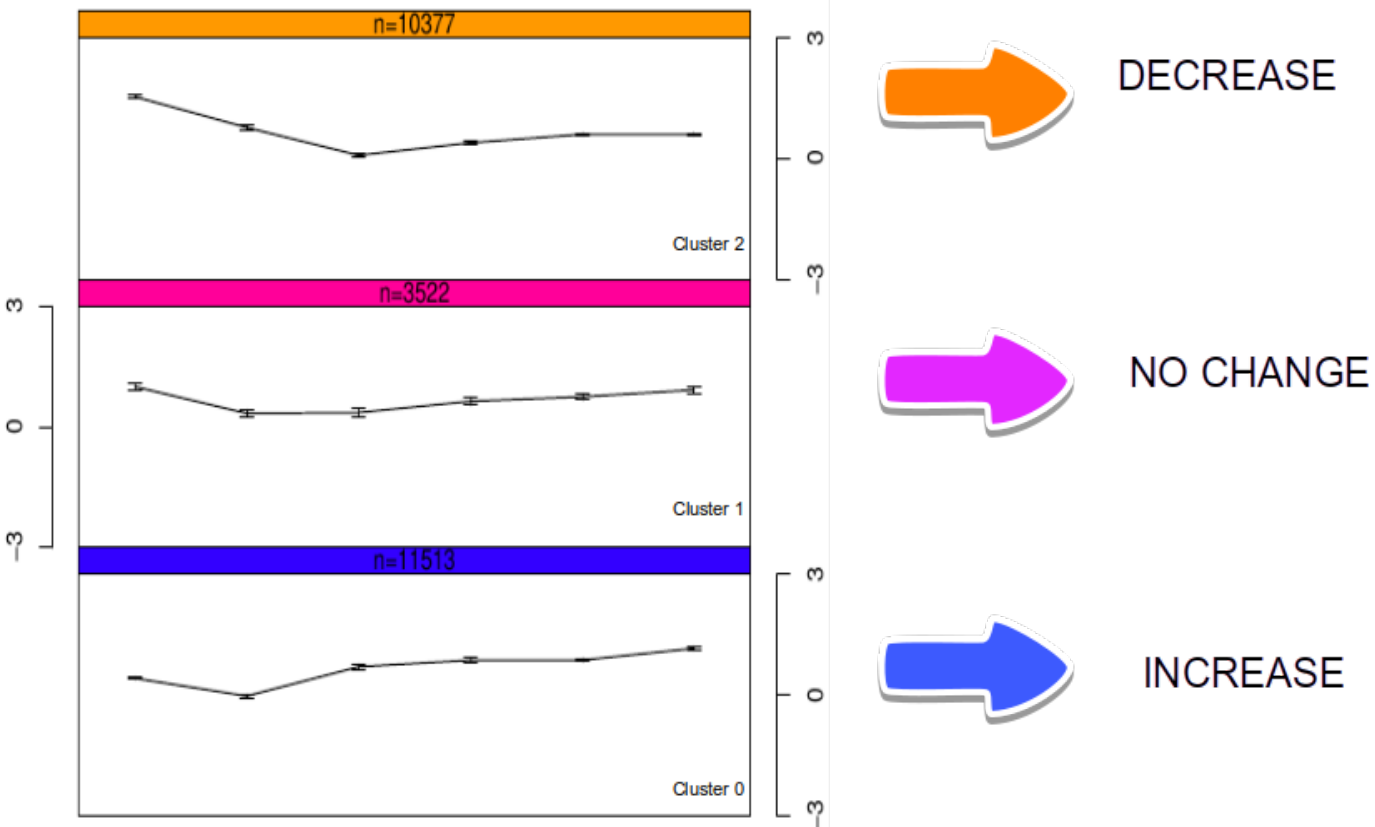

Supplement: Supplementary file 1 [file foods-15-00480-s001.zip › Figure S4.pdf]
