# Supplementary material for: Persimmon Powder from Discarded Fruits as a Potential Prebiotic to Modulate Gut Microbiota in Postmenopausal Women
Source: Foods. 2026 Jan 30;15(3):480. doi: 10.3390/foods15030480 (PMC12897050; doi:10.3390/foods15030480)
Supplement: Supplementary file 1 [file Figure S3.pdf › Figure S3.pdf]

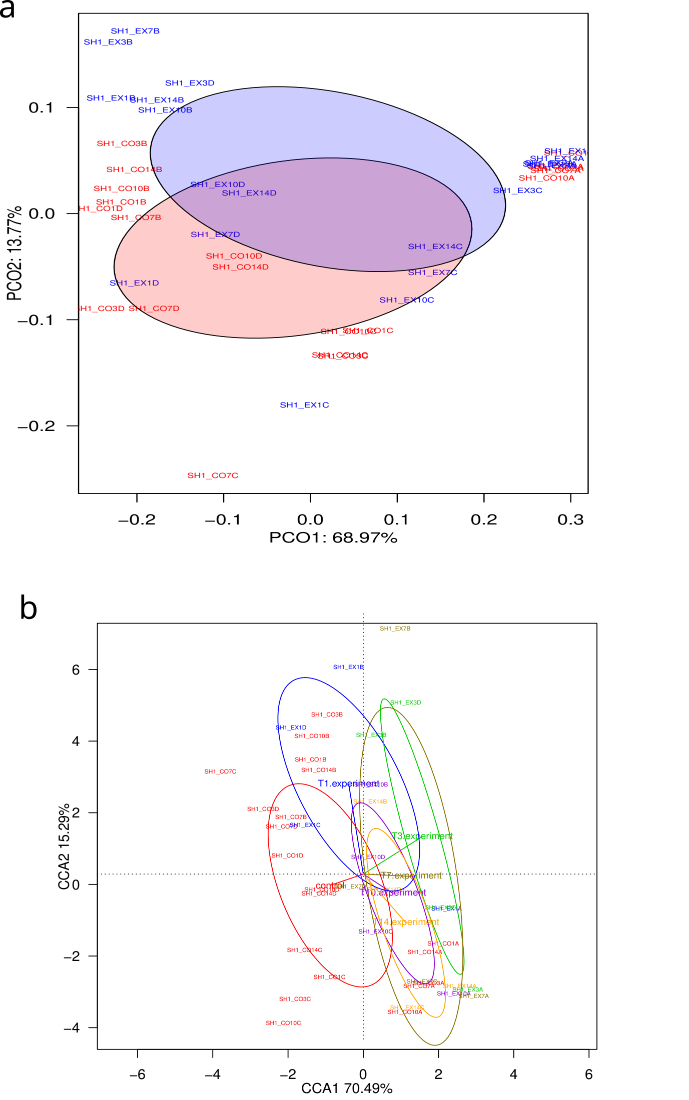
a
SH1_EX7B
SH1_EX3B
SH1_EX3D
SH1_EX1B
SH1_EX14B
SH1_EX10B
SH1_CO3B
SH1_CO14B
SH1_CO10B
SH1_CO1B
SH1_CO7B
SH1_EX10D
SH1_EX14D
SH1_EX7D
SH1_CO10D
SH1_CO14D
SH1_EX1D
SH1_CO7D
SH1_EX14A
SH1_CO10A
SH1_EX3C
SH1_EX14C
SH1_EX7C
SH1_EX10C
SH1_EX1C
SH1_CO7C
0.1
0.0
−0.1
−0.2
PCO2: 13.77%
−0.2
−0.1
0.0
0.1
0.2
0.3
PCO1: 68.97%
b
SH1_EX7B
SH1_EX1B
SH1_EX3D
SH1_CO3B
SH1_EX1D
SH1_CO10B
SH1_CO1B
SH1_CO14B
SH1_CO7C
T1.experiment
SH1_CO3D
SH1_CO7B
SH1_EX1C
SH1_CO1D
T3.experiment
T7.experiment
control
T10.experiment
T14.experiment
SH1_EX10C
SH1_CO1A
SH1_CO14A
SH1_CO14C
SH1_CO1C
SH1_CO7A
SH1_EX14A
SH1_EX3A
SH1_EX7A
SH1_CO3C
SH1_CO10A
SH1_CO10C
6
4
2
0
−2
−4
CCA2 15.29%
−6
−4
−2
0
2
4
6
CCA1 70.49%
